# Participants’ Perceptions of a Group Based Program Incorporating Hands-On Meal Preparation and Pedometer-Based Self-Monitoring in Type 2 Diabetes

**DOI:** 10.1371/journal.pone.0114620

**Published:** 2014-12-23

**Authors:** Kaberi Dasgupta, Soghra Jarvandi, Mirella De Civita, Sabrina Pillay, Samantha Hajna, Rejeanne Gougeon, Abeer Bader, Deborah Da Costa

**Affiliations:** 1 Department of Medicine, McGill University Health Centre, Montreal, Quebec, Canada; 2 School of Medicine, Division of Health Behavior Research, Washington University, St. Louis, Missouri, United States of America; 3 Community Diabetes Education Program of Ottawa, Ottawa, Ontario, Canada; Scottish Collaboration for Public Health Research and Policy (SCPHRP), United Kingdom

## Abstract

**Background:**

Nutrition education (portion sizes, balanced meals) is a cornerstone of diabetes management; however, moving from information to behavior change is challenging. Through a single arm intervention study, we recently demonstrated that combining education with group-based meal preparation training has measureable effects on weight, eating behaviour, and glycemic control in adults with type 2 diabetes. In the present study, we conducted an in-depth examination of participants’ perceptions of this strategy, through focus group discussion, to delineate effective elements of the strategy from participants’ perspectives.

**Methods:**

Participants who had completed the nutrition education/meal preparation training program were invited to attend one of four focus group discussions. These were led by experienced facilitators and guided by questions addressing experiences during the intervention and their perceived impact. Audiotapes were transcribed and qualitative content analysis of transcripts was performed. We report herein themes that achieved saturation across the four discussions.

**Results:**

Twenty-nine (80.6%, 29/36) attended a focus group discussion. The program elements perceived as effective by participants included the hands-on interactive learning approach to meal preparation, the grocery store tour, pedometer-based self-monitoring, experiencing the link between food consumption/physical activity and glucose changes during the program, and peer support. Discussants reported changes in eating and walking behaviour, greater confidence in ability to self-manage diabetes, reductions in glucose levels and/or need for glucose-lowering medications, and, in some cases, weight loss. Family members and friends were facilitators for some and barriers for others in terms of achieving health behavior changes.

**Conclusions:**

Among adults with type 2 diabetes, a group based program that included hands-on meal preparation and pedometer-based self-monitoring was perceived as effective in conveying information, developing skills, building confidence, and changing health behaviors.

## Research Highlights

1. Group meal preparation training builds knowledge, skills, and social support.
2. This enhances confidence in diabetes management and leads to eating behavior change.
3. Family engagement and support may amplify the effects.

## Introduction

The past two decades have witnessed a marked increase in the ‘outsourcing’ of food preparation. [1]–[4] Dining out and consuming prepackaged convenience foods are frequently perceived to be more labour and time-efficient. Eating out may also be an opportunity for socialization. Unfortunately, such outsourcing generally results in a dietary intake that is high in fat, starch, sugar, and overall energy intake. [5], [6] This has contributed to a rise in prevalence overweight and obesity [7], [8] and the co-epidemic of type 2 diabetes [9], [10].

A condition characterized by high blood sugar (glucose) values, type 2 diabetes results from an increase in resistance to the action of the hormone insulin. Insulin normally facilitates the entry of glucose into the cells of the body. With insulin resistance, glucose cannot gain efficient entry to function as a fuel. This causes accumulation of glucose in the blood, exacerbated further by an insulin resistance-induced stimulation of the liver to increase glucose production. Insulin resistance results from a combination of excess weight, physical inactivity, and genetic predisposition. It leads to blood vessel damage not only through direct effects of high blood glucose levels but also higher levels of blood pressure and serum lipids and other factors. Type 2 diabetes more than doubles the risk of vascular disease such as heart attack and stroke. [11], [12] Attention to eating habits may not only prevent type 2 diabetes but may also reduce the risk of its vascular complications. [13]–[15] Net weight losses of as little as 2 to 5% in persons with type 2 diabetes have been associated with improvements in both blood pressure and glucose control. [16] Achieving even such a modest weight loss, however, is challenging in an environment that promotes and facilitates the outsourcing of food preparation. [17], [18] With this in mind, we have been developing a strategy designed to promote and facilitate home-based preparation of food, emphasizing taste and time efficiency [19].

The strategy was pilot tested [19] in adults with type 2 diabetes. It consisted of 15 in-person group sessions over 24 weeks, held at the kitchen workshops of two Montreal grocery stores. Each 3-hour session involved the hands-on preparation of a healthy, balanced meal under the supervision of a chef, with concurrent education and counseling from a registered dietitian. Participants were also provided with a book of recipes endorsed by the Canadian Diabetes Association, and encouraged to prepare meals at home using this tool. They received a step counter (pedometer) to track their physical activity levels (i.e., steps per day) and encouraged to increase their daily step count progressively to 10,000 steps per day or more. Completion of an average of 10,000 steps/day is consistent with being ‘active’. [20] A previous systematic review demonstrates that pedometer-based programs lead to higher daily step counts and lower blood pressure levels in several clinical populations, particularly when a specific target is provided (e.g., 10,000 steps/day) [21].

Among the roughly three quarters of participants who completed final assessments, there were improvements in eating control (11.2 point Weight Efficacy Lifestyle Questionnaire score change, 95% CI 4.7 to 17.8) and small but conclusive reductions in weight (mean weight change –2.2%; 95% CI –3.6 to –0.8) linked to clinically- important improvement in blood glucose control (mean A1C change –0.3%, 95% CI –0.6 to –0.1), as well as a strong suggestion of improvement in systolic blood pressure (mean change –3.5 mm Hg, 95% CI –7.8 to 0.9) [19].

In this paper, we endeavour to delineate the program elements that may have led to these improvements and to identify approaches that could increase effectiveness. We report herein the results of focus group discussion analyses conducted among participants following the program. The discussions were held to better understand the participants’ impressions and experiences regarding the program’s structure and its potential effects on their long- term eating habits. Interestingly, in recent years, a number of studies have demonstrated that healthy pre-prepared meals and meal substitutes are effective weight loss tools, both in general and in type 2 diabetes [22], suggesting that ‘healthier outsourcing’ is a potential option. Notwithstanding this evidence, meal replacements and healthier prepared meals are expensive, potentially monotonous, and socially-isolating choices. It is therefore important to develop behavioural change alternatives, and to gather evidence that demonstrates effectiveness. We believe that this qualitative study provides important information regarding a novel strategy that aims to reduce vascular disease risk in type 2 diabetes.

## Methods

### Participants

Both the original intervention study [19] and the focus group component (reported herein) received approval from the Institutional Review Board of the Faculty of Medicine, McGill University, as well as from participating institutions (McGill University Health Centre, Sir Mortimer Davis Jewish General Hospital, St. Mary’s Hospital). Participants provided written informed consent. We recruited overweight adults with type 2 diabetes who were followed at McGill University-affiliated clinics or responded to poster advertisements (1 April 2009 to 8 May 2010). They were enrolled in a 24-week program (15 sessions); six series of sessions were held. Focus group study discussions occurred at the end of the final four series, at the same grocery store workshop where the intervention had been conducted.

### Discussant characteristics

We computed mean values (standard deviations) and proportions for demographic and clinical characteristics (e.g., age, sex, BMI, step counts, systolic and diastolic blood pressure) for focus group discussants and their changes in clinical variables of interest (e.g., BMI, step counts, systolic and diastolic blood pressure) during the course of the original program. We present these in S1 Information, juxtaposing changes in the discussants with the overall changes observed in the original intervention study.

### Focus Group Sessions

Each discussion lasted 120 minutes and was led by a moderator and an assistant, using questions (Table 1) developed by the research team. The questions addressed the decision to take part in the program, the actual experience of program participation, and participants’ impressions of impact on their lives. All discussions were audiotaped and transcribed (3 English, 1 French). The moderators held doctoral degrees in anthropology. Neither was involved in the original intervention study.

**Table 1:** Focus Group Interview Questions.

**Participants’ decision and challenges in taking part in the program**
a. What motivated you?
b. What challenges did you face by participating in these cooking lessons?
**Participants’ actual experience of participating in the program**
a. What did you like the most about this program?
b. What did you like (or dislike) about the lessons given by the chef/dietitian?
c. To what extent did these classes contribute to an increase in your knowledge of diabetes control?
d. In what ways was being in a group helpful?
e. What would you have improved in this program?
**Participants’ understanding of the program’s impact on their lives**
a. What changes did you make to your eating or other lifestyle habits?
b. What were the barriers/facilitators to making these changes?
c. What was the role of your family, friends, and/or relatives toward improving your lifestyle

### Analyses

A standard methodology described by Krueger [23] was adopted for qualitative content analysis of the focus group transcripts. This was performed by a clinical research health psychologist (MDC) and a health practitioner (SP) trained in qualitative methods, neither of whom was involved in the program intervention. Both independently reviewed the transcripts for an overall impression of the discussion and group dynamics, and then re-read and coded them to identify emerging themes. Text responses were classified according to which questions they addressed, and reviewed through a continuous process of comparing text segments across the groups, seeking similar or repeated ideas. Any differences in coding of text responses were discussed until agreement was reached. The next step involved labeling identified themes for each question. Several themes were identified, which were regrouped to clearly delineate the program elements that participants viewed as having impact. These are illustrated through specific quotations. French quotations have been translated into English.

## Results

### Participant Characteristics

Among the final 36 participants who completed our original intervention study [19] and were invited to participate in a focus group discussion, 29 (80.6%) participated in such a discussion. These were held in the summer and fall of 2010 (10 July 2010; 7 September 2010; 14 November 2010; 20 November 2010). Discussants (S1 Information) were middle aged to elderly, more than half were women, and approximately three quarters were of European descent. They were overweight to obese, had daily step counts (assessed with a step counter) in the low active to somewhat active range, and, on average, had type 2 diabetes for eight years. Blood sugar and blood pressure levels were somewhat above recommended targets, on average. Improvements in focus group participants during the intervention (i.e., body mass, blood sugar, blood pressure, eating habits, dietary intake) were similar to or somewhat more favourable than changes in all participants who completed baseline and final assessments (S1 Information).

### Theme 1: Effective Program Elements

Participants were able to share and comment on a number of elements that they considered to be effective in motivating them to adopt and adhere to new lifestyle choices. The elements identified, described below as subthemes, each contributed a particular influence to sustaining positive behavioral habits.

#### ‘Hands-on’ interactive learning

Discussants appreciated the interactive, hands-on learning strategy component of the program [*The hands-on approach helped to learn how to feel things differently, to chop things differently…. You retain more than if you are sent home with a bunch of papers to try at home*]. The presence of the chef and dietitian facilitated the learning process, allowing participants the time to formulate questions and comments and engage in discussion [*If you have a question and you can actually have someone answer it in that moment, the chef can show you. The nutritionist can answer a question, that’s something you see.* AND *With TV you are passive, in here you ask questions*]. Participants appreciated learning how to work with different ingredients [*It introduced me to new foods that I never heard of or seen before*]. They also commented on learning how to optimize the use of cooking utensils to derive more enjoyment from food preparation [*I’ve learned to use proper tools for cooking which makes things faster…. It’s just little tricks, new tools, which kind of make it more interesting*].

#### Grocery store tour

Some participants stressed the importance of going on the tour of a grocery store as an addition to learning about foods [*The tour of the grocery, with the dietitian was good. You learn about what to eat and what to look for*]. This offered them opportunities to ask the dietitian questions in the moment, thereby contributing to their decision-making process around food choices.

#### ‘Making the link’ of food consumption and physical activity with blood glucose levels

During the course of the program, a concrete connection that some participants were able to make concerned their understanding of how their blood sugar level was directly influenced by what they chose to eat [*My doctor used to say- eat protein with your meals. Okay I didn’t pay so much attention. But now, here when I came, the dietitian explained nicely to us how it works. I was eating protein and I saw a difference* AND *The main thing I learned is that when you have the four food groups in the right portion and you go home and take your blood sugar, it’s absolutely normal…*]. The relationship between physical activity and glucose control also became more apparent for some participants, highlighting for them the importance of an active lifestyle [*When I do exercises as I should be, my sugar is under control and I feel it. It shows on my sugar level*].

#### Support from the group

Peer support was an important facilitating element for most, minimizing a sense of isolation [*we were not alone with our problem, and we could share the problem* AND *What I liked the most was meeting people, to feel that the isolation was broken. We often find ourselves all alone with this disease and people don’t understand. Here we found ourselves with people who had similar issues such as eating too much or eating too many fat, large portions…*]. Peers were a source not only of social support but also of knowledge [*We learn from each other too. The teamwork and getting ideas from everybody*]. A few individuals expressed some discomfort with ‘working with strangers’ and coordinating meal preparation with others.

#### Pedometers

Pedometers were generally perceived as being helpful in motivating participants to engage in walking [*For me it was a nice gadget. I tried to do better every time, you know*]. For some, the use of the pedometer enhanced their awareness of current physical levels, with some pleased [*I found out that it’s a lot of steps – cleaning the house. A lot more than I expected*] and others disappointed [*I had the impression I was walking miles and miles…I realized that a weekend of cleaning, doing many things, was wow, completely the opposite…the pedometer helped me, in a sense, to measure. It is a tool I will use continuously…*]. Those participants who expressed dissatisfaction with the pedometer mostly alluded to modifiable technical difficulties.

### Theme 2: Changes Attributed to the Program

Participants described how they consciously made changes as they continued to attend program sessions. For some, the changes highlighted related to behaviors, whereas for others it was further evidenced in terms of weight loss, greater self-confidence in managing diabetes, and better glucose control without medications. Two subthemes emerged that describe the changes participants strongly believed could be attributed to the program experience.

#### Health behaviours

Participants noted more regular meals [*I never used to have breakfast*], greater intake of healthier foods [*I take more vegetables* AND *I have a lot more fish at home*], and being more selective with foods when shopping [*I would look at the labels…* AND *I read labels better*], and dining out [*Before I used to go to go to many restaurants where there are buffets, you know… But since I’ve been coming here, I’ve stopped going to those places.* AND *When we eat out it is different. You make different choices*]. A few participants, across all four groups, described how the program helped them to incorporate more walking [*A lot of walking and I’m addicted to walking now…*AND *It motivated me to walk more…*].

#### Weight change

With respect to weight loss, some participants were very satisfied [*We become more conscientious of what we should be doing. I began paying attention to my food quantity. Watch my food portions because in reality my diet didn’t change all that much. However, the portion sizes did, and I began to lose weight naturally*], and others less so [*…not as much as I may have wanted to, but it did go down a little*]. More consistently, however, there was greater confidence in diabetes management, improvements in glucose control, and reductions in medications.

#### Greater self-confidence in ability to manage diabetes

Some made direct reference to the program improving their self-confidence to manage diabetes as part of their lives [*…we learnt how to live with diabetes and not let the diabetes prevent us from living…what’s important is our disease and being confident with regards to the disease*]. Others were delighted in having acquired new culinary skills and spoke of the impact it had on their confidence, [*…this gave me a gastronomical independence…it gave me the taste and confidence that I could prepare something in the kitchen and me, who has never, ever, done anything in the kitchen, started cooking.*]. For the participants already skilled in cooking, the program helped to confirm their knowledge and encourage good habits [*What I really liked about the lessons is that it gave me more confidence in myself, it made me realize that yes, you can go to these courses and you can have an opinion of your own, and yours isn’t that bad*].

#### Better glucose control and reductions in medication use

Some participants expressed satisfaction with the program in terms of reduction in need for glucose-lowering medications [*I no longer take medications since I’m here* AND *I am more and more without medication*]. Others referred to improvements in glucose control [*with this program, I am well controlled*] as a health outcome. While some still struggled with attaining ideal glucose levels, it was recognized that this program was helpful in attempting to reach this goal [*Oh yeah, absolutely. Helped me cut down my sugar immensely. I still have problems regulating my numbers. I mean, but that’s just a personal thing. I go crazy every now and then…*].

### Theme 3: Family Members and Friends as potential facilitators and barriers

The most important challenge to participation identified by participants was family-related responsibility [*I had to return quickly home to cook her* (mother with disability) *dinner*. AND *I have to cook for him* (husband) *before I go to the program* AND *Once school started, things changed. I could no longer attend as I used to*].

Some women found it challenging to change their diet because of their husbands, […*it’s hard to give falafel to him, let’s say. He wouldn’t like it.* AND *I live with my husband who is very difficult, he wants only meat, potatoes and rice, and stuff like that*]. It appeared to be easier for men to make these changes, having spouses who were willing to facilitate the process, compared to women who participated in the program [*Well, for me it’s very easy, my wife, she’s doing everything. (Laughing) So I give her everything I learnt in the course, and she tries to apply it*].

Many participants drew strength from their children in maintaining changes in health behaviours [*…my kids noticed that I’m kind of watching my steps and everything. So like this weekend…after supper, one of them looks at me and says, ‘it must be low today Mom, let’s go for a walk.’ So they just kind of help in a way.*]. Others noticed how the benefits of the program trickled down to their children [*…actually, one of my sons has lost weight. He’s down to what he was when he was much younger. He’s not eating the salty potato chips and other things that he used to. So he’s taken an example from me, ‘If mother can do it, so can I*’].

Friends were also identified as playing a role in either helping participants to make and sustain better lifestyle choices or influencing negative behaviours. Participants specifically described how their friends were helpful in sustaining healthier choices by making compromises themselves [*They really make food that I can eat. If I’m invited somewhere, nobody serves me just pasta*]. Still, for a few participants in our groups, their peer group made it more difficult to maintain healthy eating habits [*I’m 71 and I joined every seniors’ group in Montreal and all they do is eat!*].

### Theme 4: Improvements to the Program: Need for Tracking and Monitoring

Participants stressed the importance of having an objective approach to capturing the extent of knowledge acquired [*What can be improved is if we have a question and answer form. If we have forms with questions and answers, we become more conscious about what it’s all about*]. Others expressed interest in being able to track what they were doing [*The courses make us conscious…. Maybe if we could also have a calorie sheet so that way we become more conscious of what we put in our mouths, not just the walking part*].

## Discussion

**Figure 1:**
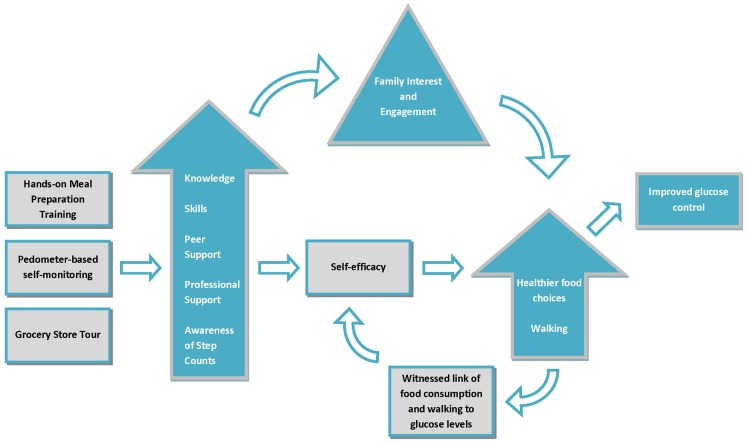
Schematic summary of focus group themes and their possible inter-relationships. The program tested included group-based hands-on meal preparation training under the supervision of a dietitian and chef, a grocery store tour, and pedometer-based self-monitoring. Focus group analyses indicate that the program led to a perception of peer and professional support, greater skills in meal preparation, more knowledge of healthy food choices and combinations, and greater awareness of step counts through pedometer-based self-monitoring. These stimulated changes in eating and physical activity behaviours and witnessing of impact on glucose levels. This enhanced self-efficacy in terms of diabetes management. In many cases, program participation stimulated health-related discussion at home; family interest and engagement enhanced the program’s effects. Lack of such engagement from family or friends, however, posed a barrier to improvement for some.

Glucose control is a central focus of type 2 diabetes management. Changes in eating and physical activity behaviours can importantly facilitate blood glucose lowering, reducing reliance on medication and concurrently improving other vascular risk factors such as elevated blood pressure. In a previous analysis, [19] we demonstrated that a group-based program with hands-on meal preparation training and pedometer-based self-monitoring could importantly lower glucose levels in adults with type 2 diabetes, even without marked reductions in weight. In the present qualitative analysis, we have delineated the program elements that the participants perceived as leading to this improvement (Fig. 1). These included the hands-on, interactive learning approach to meal preparation, the grocery store tour, pedometer-based self-monitoring, and peer support. During the program, they were able to ‘live’ the connection between improvements in eating and physical activity behaviours and improvements in blood glucose control. While they had been informed of this link in the past, the regular interactions with the chef, dietitian, and peers motivated them to adopt behavior changes, and they then witnessed the impact of these behaviours.

The specific changes that participants reported included more regularity in meal consumption, such as not skipping breakfast, greater intake of vegetables and fish, reflection on food choices when shopping and eating out, and higher levels of walking. While the impact on weight was variable, participants more consistently observed reductions in their glucose levels and/or in doses of glucose-lowering medications. Family members were identified as both barriers and facilitators to program participation and adoption of healthy nutritional habits. Specifically, family-related responsibilities and taste preferences could pose challenges to changing eating habits, but some family members provided encouragement and support. Improvements in the dietary and physical activity habits of other family members were also motivating.

Social support emerged as a critical element to effectiveness of the strategy, consistent with the findings of a recent systemic review which identified social support as a key component associated with increased change in diet and/or physical activity in individuals at risk of type 2 diabetes. [15] The peer group reduced a sense of isolation in managing type 2 diabetes. The patience and availability of the chef and dietitian were greatly appreciated, perhaps in contrast to the brevity of many clinical encounters. Thus the shared activity of preparing and consuming a balanced meal had a motivating influence. Home-based social support, in the form of encouragement from family members, was also highly valued and motivating, particularly when family members also altered their eating and activity habits. Correspondingly, resistance of other family members to changes in meals was a potential barrier to change. This suggests that other family members may need to be more explicitly engaged in the behavioural change process, perhaps through participation in some sessions.

The nutritional information and culinary skills- building were also important elements. These led to greater consciousness of eating habits and consciousness of eating patterns. This in turn resulted in improved glucose control and/or weight change, further reinforcing the changes in habits. This is consistent with the Common Sense Model of Self- Regulation [24], which suggests that adherence to healthy behavior recommendations is facilitated by personal experience of positive change. This is also in line with the construct of testability in diffusion of innovations theory [25].

Interestingly, participants voiced a need for higher levels of tracking and monitoring, although they were tracking step counts with a pedometer (step counter) and each session commenced with a weigh-in. They appeared to desire a more personalized approach to goal- setting and accountability. This is consistent with our own work examining experiences during and following a supervised exercise program. [26] There is some evidence that self- monitoring of intake improves adherence to nutritional education, and digital technology may facilitate such monitoring [27]–[29] Review of such records, however, may be more challenging in a group-based context, and suggests a need to couple a group-based program with individualized review of self-monitoring records, perhaps during routine medical follow-up. While medical follow-up visits may not lend themselves to comprehensive behavioural change interventions, they may prove to be useful as sources of accountability and monitoring. Another alternative is feedback and support delivered through Internet-based sites linked with web-based logs. There is an emerging body of evidence suggesting promising results for Internet based features (i.e., on-line progress charts, journals) and social support (i.e., web chats) in terms of impact on weight loss and maintenance following program completion [30].

Participants expressed that the intervention increased their self- confidence (self-efficacy) in their ability to improve nutritional intake and manage diabetes. Self-efficacy, a construct from Social Cognitive theory that refers to one’s confidence to perform a given behaviour, [31] has previously been shown to be a key element in effective behaviour change diabetes prevention programs. [32] This finding lends support to the importance of including theory- driven behaviour change techniques to maximize the effectiveness of diabetes prevention programs [15], [32].

The focus group discussants included only those who had persisted with the program, not those who had been unable to continue. We acknowledge this as a limitation; the views of those who did not persist with the program may have differed. It is clear, for example, that on average focus group participants had more favourable outcomes in terms of weight loss, glycemic control, and blood pressure (S1 Information). Nonetheless, our qualitative findings, as identified through focus group discussion analyses, provide supporting evidence for the role that the intervention played in improving glycemic control and reducing body weight, as well as increasing step counts; that is, the themes that emerged from the focus group discussions indicate that the pre/post intervention improvements that we measured (i.e., weight reduction, step count increase, improved eating behavior control, hemoglobin A1c reduction, blood pressure improvements) [19] were indeed attributable to key components of the intervention itself, through its effects on knowledge, skills, and behavior.

Improving dietary and physical activity habits is a challenge for a large proportion of the population in the 21^st^ century. Adults with type 2 diabetes constitute a group of individuals who have not only developed their condition as a result of suboptimal eating and physical activity habits but are also at high risk of vascular disease-related complications, particularly if these health behaviours are not optimized. Conversely, small changes may result in important effects. The words and perspectives of our participants indicate that important elements to achieving behavioural change include not only knowledge, but also social support, skills-building, practice, and monitoring. The development and availability of programs aiming to provide this may be critical in stemming the tide of vascular disease complications that may be linked to the obesity and diabetes co-epidemics.

## Supporting Information

S1 Information **Table S1.** Socio-demographic Characteristics, Clinical Measures, and Medications at Baseline. **Table S2.** Step Counts, Dietary Intakes, and Psychobehavioural Characteristics at Baseline. **Table S3.** Changes and 95% Confidence Intervals from baseline to the final assessment.
(DOCX)
