# Supplementary material for: Participants’ Perceptions of a Group Based Program Incorporating Hands-On Meal Preparation and Pedometer-Based Self-Monitoring in Type 2 Diabetes
Source: PLoS One. 2014 Dec 23;9(12):e114620. doi: 10.1371/journal.pone.0114620 (PMC4275207; doi:10.1371/journal.pone.0114620)
Supplement: S1 Information — Table S1. Socio-demographic Characteristics, Clinical Measures, and Medications at Baseline. Table S2. Step Counts, Dietary Intakes, and Psychobehavioural Characteristics at Baseline. Table S3. Changes and 95% Confidence Intervals from baseline to the final assessment. (DOCX) [file pone.0114620.s001.docx]

| **Table S1. Socio-demographic Characteristics, Clinical Measures, and Medications at Baseline.** | | |
| --- | --- | --- |
|  | **Final Assessment Attended**  **(n=53)^[[1]](#footnote-1)^** | **Focus Group Participants**  **(n=29)^[[2]](#footnote-2)^** |
| Socio-demographic Characteristics |  |  |
| Age, years, Mean (SD) | 59.7 (10.6) | 58.9 (11.3) |
| Women, No. (%) | 36 (67.9) | 19 (65.5) |
| Europid, No. (%) | 40 (75.5) | 24 (82.8) |
| Born in Canada, No. (%) | 27 (50.9) | 15 (51.7) |
| Ever smoked, No. (%) | 27 (50.9) | 16 (55.2) |
| Income < $50,000, No. (%) | 29 (56.9) | 16 (59.3) |
| Employed, No. (%)^[[3]](#footnote-3)^ | 27 (50.9) | 13 (44.8) |
| Education (>high school), No. (%) | 42 (79.3) | 23 (79.3) |
| Living Alone, No. (%) | 19 (35.9) | 11 (37.9) |
|  |  |  |
| Clinical Measures |  |  |
| Weight, kg, Mean (SD) | 88.3 (14.6) | 92.5 (14.6) |
| BMI, kg/m^2^, Mean (SD) | 33.1 (5.0) | 34.7 (5.0) |
| Systolic blood pressure, mm Hg, Mean (SD) | 133.4 (12.5) | 135.5 (13.2) |
| Diastolic blood pressure, mm Hg, Mean (SD) | 80.4 (9.0) | 83.1 (8.5) |
| HbA1c, %, Mean (SD) | 8.0 (1.7) | 8.1 (1.5) |
| Years with diabetes, Mean (SD) | 8.5 (8.7) | 8.1 (8.4) |
|  |  |  |
| Medication Use |  |  |
| *Antihypertensive agents, No. (%)* |  |  |
| One | 14 (26.9) | 9 (32.1) |
| Two | 9 (17.3) | 3 (10.7) |
| More than two | 11 (21.2) | 4 (14.3) |
| *Antihyperglycemic agents, No. (%)* |  |  |
| One oral Agent | 22 (42.3) | 13 (46.4) |
| Two oral Agents | 14 (26.9) | 8 (28.6) |
| More than two oral agents | 4 (7.7) | 2 (7.1) |
| Insulin | 13 (25.0) | 6 (21.4) |

| **Table S2. Step Counts, Dietary Intakes, and Psychobehavioural Characteristics at Baseline.** | | |
| --- | --- | --- |
|  | **Final Assessment Attended**  **(n=53)^[[4]](#footnote-4)^** | **Focus Group Participants**  **(n=29)** |
| Step Counts, steps/day, Mean (SD) | 7,272 (2,777.0) | 7,945 (2,972.6) |
|  |  |  |
| *Dietary Intake^[[5]](#footnote-5)^* |  |  |
| Energy, kcal/day, Mean (SD) | 2,024 (839) | 2,000 (757) |
| Protein, % of total energy intake, Mean (SD) | 19.2 (3.7) | 20.9 (5.1) |
| Carbohydrate, % of total energy intake, Mean (SD) | 44.3 (6.8) | 42.9 (7.3) |
| Fat, % of total energy intake, Mean (SD) | 36.7 (6.1) | 36.5 (5.8) |
| Saturated fat, % of total energy intake, Mean (SD) | 11.1 (2.3) | 10.9 (1.8) |
| Fibre, g/day, Mean (SD) | 21.2 (10.3) | 20.2 (8.7) |
| Sodium, mg/day, Mean (SD) | 2,903 (1,255) | 2,966. (1,246) |
| Alcohol Consumption (< once per week), No. (%) | 43 (81.1) | 22 (75.9) |
|  |  |  |
| *Weight Efficacy Life-Style Scores*, *Mean (SD)^[[6]](#footnote-6)^* |  |  |
| Availability Subscale | 20.3 (7.8) | 20.8 (7.3) |
| Negative Emotions Subscale | 20.3 (9.1) | 19.8 (9.8) |
| Physical Discomfort Subscale | 25.4 (7.3) | 26.0 (7.2) |
| Positive Activities Subscale | 25.1 (7.6) | 25.7 (7.6) |
| Social Pressure Subscale | 23.9 (7.5) | 24.8 (7.1) |
| Total WEL Score | 115.0 (32.9) | 117.1 (34.1) |
|  |  |  |
| *Hours/day spent cooking and washing up, Mean (SD)* |  |  |
| Weekdays | 1.5 (1.0) | 1.2 (0.9) |
| Weekend days | 1.7 (1.2) | 1.7 (1.2) |
|  |  |  |
| Self-perception as competent cook, No. (%)^[[7]](#footnote-7)^ | 21 (39.6) | 10 (34.5) |
| Frequency of eating out (days/week), Mean (SD) | 1.9 (1.8) | 2.0 (2.0) |
|  |  |  |
| *Stage of Change for Weight Change Efforts,*  *No. (%)* |  |  |
| Pre-contemplation | 2 (3.8) | 1 (3.5) |
| Contemplation | 13 (24.5) | 5 (17.2) |
| Action | 29 (54.7) | 20 (69.0) |
| Maintenance | 9 (17.0) | 3 (10.3) |
|  |  |  |
| Depressive Symptomology (CES-S score >16), No. (%) | 15 (28.3) | 10 (34.5) |

| **Table S3. Changes and 95% Confidence Intervals from baseline to the final assessment.** | | |
| --- | --- | --- |
|  | **Final Assessment Attended**  **(n=53)^[[8]](#footnote-8)^** | **Focus Group Participants**  **(n=26)^[[9]](#footnote-9)^** |
| **Changes in Clinical Measures** |  |  |
| ≥5% weight loss, % | 18.9 (9.4, 32.0) | 34.6 (17.2, 55.7) |
| Percent weight change | -2.2 (-3.6, -0.8) | -4.5 (-6.9, -2.1) |
| BMI, kg/m^2^ | -0.9 (-1.3, -0.4) | -1.7 (-2.5, -0.9) |
| Systolic blood pressure, mm Hg | -3.5 (-7.8, 0.9) | -6.7 (-13.6, 0.1) |
| Diastolic blood pressure, mm Hg | -0.1 (-3.0, 2.8) | -3.5 (-8.1, 1.1) |
| HbA1c, % | -0.3 (-0.6, -0.1) | -0.6 (-1.0, -0.1) |
|  |  |  |
| **Changes in Dietary Intakes** |  |  |
| Total energy, kcal/day | -239.(-408, -70) | -214 (-478, 50) |
| Protein, % of total energy intake | 0.8 (-0.2, 1.8) | 0.7 (-0.9, 2.4) |
| Carbohydrate, % of total energy intake | -2.1 (-4.2, -0.1) | -1.8 (-5.3, 1.8) |
| Fat, % of total energy intake | 1.2 (-0.8, 3.2) | 0.2 (-3.0, 3.4) |
| Saturated fat, % of total energy intake | 0.5 (-0.2, 1.3) | 0.8 (-0.3, 1.9) |
| Fibre, g/day | -1.8 (-3.8, 0.2) | -1.1 (-4.6, 2.4) |
| Sodium, mg/day | -384 (646, -122) | -436.5 (-869.8, -3.1) |
|  |  |  |
| **Changes in Step Counts** |  |  |
| Step Counts, steps/day | 869 (198, 1540) | 965 (3.0, 1,927) |
|  |  |  |
| **Changes in Psychobehavioural Characteristics** |  |  |
| *Weight Efficacy Life-Style Scores^[[10]](#footnote-10)^* |  |  |
| Availability Subscale | 2.3 (0.4, 4.2) | 2.7 (0.2, 5.1) |
| Negative Emotions Subscale | 2.7 (0.9, 4.5) | 2.7 (0.01, 5.3) |
| Physical Discomfort Subscale | 2.1 (0.6, 3.6) | 1.8 (-0.6. 4.2) |
| Positive Activities Subscale | 2.3 (0.8, 3.7) | 1.2 (-0.8, 3.1) |
| Social Pressure Subscale | 1.9 (0.3, 3.6) | 1.9 (-0.4, 4.2) |
| Total WEL Score | 11.2 (4.7, 17.8) | 10.2 (0.5, 19.9) |
| *Hours/day spent cooking and washing up* |  |  |
| Weekdays | 0.5 (0.03, 1.0) | 1.1 (0.4, 1.8) |
| Weekend days | 0.04 (-0.3, 0.4) | -0.04 (-0.5, 0.5) |
| Frequency of eating out (days/week) | 0.1 (-0.3, 0.4) | 0.4 (-0.2, 1.0) |

1. Income was based on a sample size of 51; Years with diabetes was based on a sample size of 51; Medication use data were based on a sample size of 52. [↑](#footnote-ref-1)
2. Income was based on a sample size of 27; Years with diabetes was based on a sample size of 27; Medication use data were based on a sample size of 28. [↑](#footnote-ref-2)
3. Compared to individuals who are unemployed or on sick leave. [↑](#footnote-ref-3)
4. Steps counts per day were based on a sample size of 52 and represented the average steps per day taken during weeks 1 to 3; Frequency of eating out (days/week) was based on a sample size of 48 and represented the average frequency of eating out during weeks 1 to 3. [↑](#footnote-ref-4)
5. Assuming that 1 gram of protein equals 4 kcal, 1 gram of carbohydrates equals 4 kcal, 1 gram of total fat equals 9 kcal and 1 gram of saturated fat equals 9 kcal. [↑](#footnote-ref-5)
6. A higher score is indicative of a greater ability to resist eating under the given condition. WEL subscale scores range from 0 to 36. The Total WEL Score ranges from 0 to 180. [↑](#footnote-ref-6)
7. Compared to individuals who perceived that they can cook with a recipe, can prepare simple meals, it is not their role to cook or do not know how to cook [↑](#footnote-ref-7)
8. Change in HbA1c was based on a sample size of 51; Change in step counts was based on a sample size of 43 and represented the difference between the average steps per day taken during weeks 13 to 15 and the average steps per day taken during weeks 1 to 3. [↑](#footnote-ref-8)
9. Change in HbA1c was based on a sample size of 25; Change in frequency of eating out was based on a sample size of 17. [↑](#footnote-ref-9)
10. A higher score is indicative of a greater ability to resist eating under the given condition. WEL subscale scores range from 0 to 36. The Total WEL Score ranges from 0 to 180. [↑](#footnote-ref-10)
